# Rest and relaxation may be the key to more effective sea turtle conservation

**DOI:** 10.1093/conphys/coaa006

**Published:** 2020-02-28

**Authors:** Essie M Rodgers

**Affiliations:** Division of Ecology and Evolution, Research School of Biology, The Australian National University, 46 Sullivans Creek Road, Acton, Australian Capital Territory 2601, Australia

Stranded sea turtles are frequently rescued, rehabilitated and readied for release, but these animals must overcome one final hurdle before returning to the wild. Turtles must endure long journeys, typically in cars or vans, to reach coastal release sites. These trips are no walk in the park—or swim in the ocean—for turtles, however. Journeys can exceed 12 hours and can include cramped conditions, unfamiliar sounds, vibrations and unpredictable acceleration patterns. But, it turns out, if the turtles have time to rest and relax after their long journey, they can return to the wild much less stressed.

Travel triggers a physiological stress response in turtles that is characterised by a spike in adrenal hormones (e.g. corticosterone, cortisol and catecholamines), altered immune function and a suppression of non-essential processes, like digestion. This heightened state of stress can be harmful, particularly when experienced for prolonged periods of time. Animals released into the wild immediately following long car trips can be more vulnerable to predators and disease, and they may even flee from the release site.

Innovative turtle release practices may be the key to overcoming transport-related stress. Dr Kathleen Hunt and her colleagues (2019) tested the effectiveness of ‘soft release’ techniques. Sea turtles were transported in cars—a 24-hour-long journey—to release sites, held in saltwater pools for either 6 or 24 hours and then released into the wild. The researchers hoped that the saltwater recovery pools would be an ideal refuge for turtles to rest, relax and recover from the arduous car trip.

The team examined 18 Kemp’s ridley sea turtles (*Lepidochelys kempii*), the world’s most endangered sea turtle species. These turtles had been rehabilitated at the New England Aquarium’s sea turtle center in Boston, MA. The turtles were then transported hundreds of kilometres, by car, to coastal Georgia for release. To test the effectiveness of the saltwater pools in facilitating recovery, small blood samples were obtained before transport, immediately after transport and following 6 and 24 hours in saltwater recovery pools. The blood samples allowed the researchers to track a suite of stress and immune system markers in the turtles.

As expected, the long road trip triggered a physiological stress response in the turtles. Stress hormone levels (i.e. corticosterone) were five times higher than pre-trip levels! Glucose and potassium levels were disrupted, and immune responses were heightened. After turtles spent 6 hours in the saltwater recovery pools, corticosterone levels had partially recovered and glucose and potassium levels had returned to healthy, pre-transport levels. Prolonging recovery time to 24 hours did not further benefit the turtles, indicating that a 6-hour recovery period was sufficient to prepare them for release.

Providing turtles with a safe space to get some much-needed rest and relaxation appears to be key in helping turtles cope with the final hurdle of a long road trip. This solution could have huge benefits towards the conservation of turtles, at least until business class flights become available on their favourite airlines.

Illustration by Erin Walsh; ewalsh.sci@gmail.com


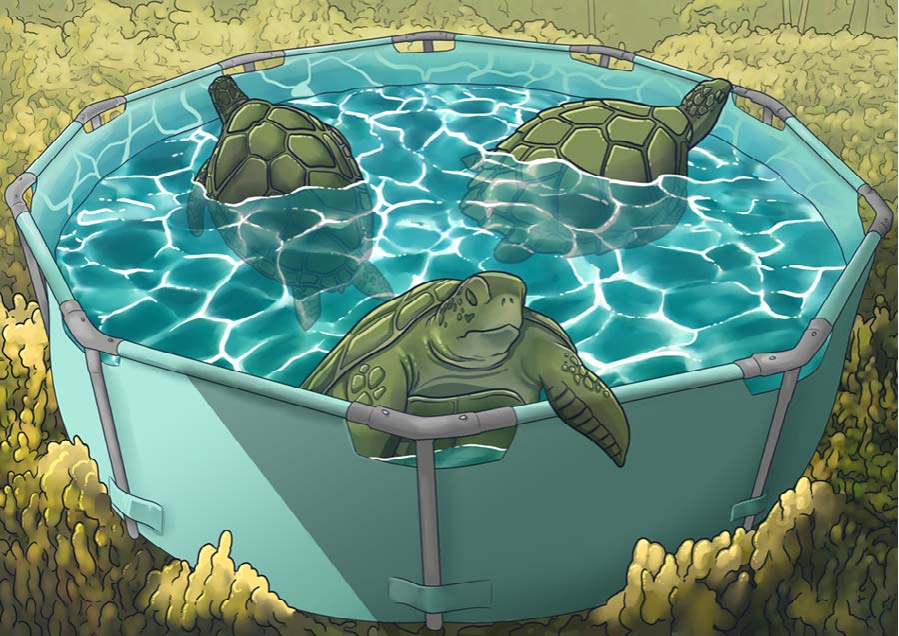


## References

[ref1] Hunt KE, Innis C, Merigo C, Burgess EA, Norton T, Davis D, Kennedy AE, Buck CL (2019) Ameliorating transport-related stress in endangered Kemp’s ridley sea turtles (Lepidochelys kempii) with a recovery period in saltwater pools. Conserv Physiol 8 1 (1): coy065. doi:10.1093/conphys/coy065. PMC6312763 30619610

